# SAR Ground Moving Target Indication Based on Relative Residue of DPCA Processing

**DOI:** 10.3390/s16101676

**Published:** 2016-10-12

**Authors:** Jia Xu, Zuzhen Huang, Liang Yan, Xu Zhou, Furu Zhang, Teng Long

**Affiliations:** 1 Beijing Key Laboratory of Embedded Real-time Information Processing Technology, School of Information and Electronics, Beijing Institute of Technology, Beijing 100081, China; hzzhit@126.com (Z.H.); yanliang121@126.com (L.Y.); zhouxu@bit.edu.cn (X.Z.); longteng@bit.edu.cn (T.L.); 2 Shanghai Aircraft Manufacturing Co., Ltd., Shanghai 200436, China; zhangfuru@comac.cc

**Keywords:** synthetic aperture radar (SAR), ground moving target, displace phase center antenna (DPCA), relative residue, river surface

## Abstract

For modern synthetic aperture radar (SAR), it has much more urgent demands on ground moving target indication (GMTI), which includes not only the point moving targets like cars, truck or tanks but also the distributed moving targets like river or ocean surfaces. Among the existing GMTI methods, displaced phase center antenna (DPCA) can effectively cancel the strong ground clutter and has been widely used. However, its detection performance is closely related to the target’s signal-to-clutter ratio (SCR) as well as radial velocity, and it cannot effectively detect the weak large-sized river surfaces in strong ground clutter due to their low SCR caused by specular scattering. This paper proposes a novel method called relative residue of DPCA (RR-DPCA), which jointly utilizes the DPCA cancellation outputs and the multi-look images to improve the detection performance of weak river surfaces. Furthermore, based on the statistics analysis of the RR-DPCA outputs on the homogenous background, the cell average (CA) method can be well applied for subsequent constant false alarm rate (CFAR) detection. The proposed RR-DPCA method can well detect the point moving targets and distributed moving targets simultaneously. Finally, the results of both simulated and real data are provided to demonstrate the effectiveness of the proposed SAR/GMTI method.

## 1. Introduction

As an all-day, all-weather and high-resolution modern sensor, synthetic aperture radar (SAR) has been widely used in many military and civilian applications. Multifunctional SAR with large-area static scene imaging and ground moving target indication (SAR/GMTI) has drawn much more attentions in recent past decades [1,2,3,4,5,6,7,8,9,10,11,12,13,14,15,16,17,18,19,20,21,22,23,24,25,26,27,28,29,30,31,32]. In the most of applications, not only the point moving targets but also the distributed moving targets are interested. The point moving targets have small size and only occupy several pixels in the conventional SAR images with meter-level spatial resolution, such as cars, trucks and tanks. The distributed moving targets normally occupy plenty of pixels in the SAR images, like the rivers and ocean surfaces. With more and more generated SAR images with complicated background, they contain not only the small-sized ground moving targets but also the large-sized moving component like rivers and ocean currents. Therefore, in many real SAR/GMTI applications it is a natural problem can we detect these moving targets or components with different sizes simultaneously?

For the single channel SAR system, the Doppler center and Doppler modulation differences are utilized for GMTI between the moving targets and the background clutter [4,5,6,7]. However, their performance will decrease remarkably when the target’s spectrum is submerged in the clutter, which is very common for slowly moving targets or SARs with fast moving platforms. Then some improved methods have been proposed to address this problem in a single channel system. In [8], a virtual multichannel is obtained based on an actual single channel system, which can detect the slowly moving targets. However, it requires a high pulse repetition frequency (PRF), which may be contradict to the large-swath applications. Therefore, many multichannel SAR methods have been proposed, such as displaced phase center antenna (DPCA) [1,9,10], along track interferometry (ATI) [11,12,13,14], space-time adaptive processing (STAP) [15,16,17,18] and velocity synthetic aperture radar (VSAR) [19,20,21,22]. Furthermore, a method combining SDAP and ISAR technique is proposed in [23] to improve the detection performance. In term of the dual-channel SAR system, ATI and DPCA are the two most popular techniques for GMTI. Both of them exploit the differences on the echoes of two along-track channels for observing the same scene with small time difference. After co-registration and channel balancing, the ATI technique detects the targets based on the interferometric phase, while the DPCA technique utilizes energy residue by subtraction of the two channels’ outputs. The ATI is suitable for the targets with large size, e.g., river and ocean current measurements [24,25], and its effectiveness has been demonstrated in SRTM [25] and TerraSAR-X [26]. However, without effective clutter cancellation, the detection performance of ATI for the point moving targets is normally unsatisfactory. In this regard, Gierull analyzes the relationship between interferogram magnitude and phase in [13], and he proposed a two-step detector for ground moving targets [27]. Nevertheless, due to the influences of “phase excursion” and speckle fluctuation noise, the detection threshold is hard to determine for point moving targets. Compared to ATI, DPCA may obtain better detection performance for point moving targets by introducing the cancellation among two channels’ complex-valued images with fixed or adaptive weights. However, because the cancellation response of DPCA filter is constant for certain Doppler, the GMTI performance of DPCA depends on the target’s radial velocity and original signal-to-clutter ratio (SCR). For the weak moving targets with low SCR, they are difficult to be detected because the absolute residue of the weak targets is still far lower than that of ground clutter. Take the river surfaces for example, they are always dim in the SAR image because of specular scattering, so their DPCA absolute residues are difficult to be detected in the strong ground background via conventional constant false alarm rate (CFAR) detection, although they are moving and sustain much relative energy after DPCA cancellation. Obviously, neither ATI nor DPCA can well detect the point moving targets and distributed moving targets, simultaneously.

Based on the existing DPCA method, this paper proposes a novel GMTI method called relative residue of DPCA (RR-DPCA). Different from the existing DPCA processing, not the absolute residues but the relative residues are used for the subsequent CFAR detection. After conventional DPCA processing in the image domain, the multi-look images of dual channels are calculated to normalize the original DPCA outputs. Based on the proposed division operator, the residue of weak component will be normalized with small denominator while the residue of strong target will be normalized with large one, which is just the cause for the name of “RR-DPCA”. The proposed RR-DPCA may be more sensitive for target’s motion than DPCA. Furthermore, because the rivers and oceans have large size and always move slowly, their shift phenomenon [28] is not obvious in the image domain, so the pixels of them may not contain ground clutter, and their SCR will be remarkably increased by the proposed RR-DPCA processing. In this paper, the statistical distribution after RR-DPCA is further analyzed, it is found that they obey Rayleigh distribution for the homogenous background, and the cell average (CA) method can be well applied for subsequent constant false alarm rate (CFAR) detection. It is shown that the proposed RR-DPCA can detect the point moving targets and distributed moving targets like river surfaces, simultaneously. Finally, the results of both simulated and real data are provided to demonstrate the effectiveness of the proposed method.

The remainder of this paper is arranged as follows. In Section 2, the performance of DPCA is analyzed and the RR-DPCA is proposed. In Section 3, the statistical distribution and the influence of window size in RR-DPCA processing is discussed, then the flowchart is given for the proposed method. In Section 4, the simulated and real data results are provided to demonstrate the effectiveness of the proposed method. In Section 5, some conclusions are drawn.

## 2. The Proposed RR-DPCA Method

### 2.1. DPCA Processing

At first, the echoes collected by dual-channel SAR are focused by certain imaging algorithm, e.g., range-Doppler (RD) algorithm, Chirp-scaling (CS) algorithm and so on [26]. Subsequently, two SAR images are co-registrated and channel balanced effectively. That is, the same ground patch is located at the same pixel on the two images, and the response differences are effectively compensated between two channels on amplitude and phase. Then, the DPCA outputs can be obtained as

(1) $$z_{\text{DPCA}}\left(m,n\right)=\left|x_{1}\left(m,n\right)-x_{2}\left(m,n\right)\right|$$

where $x_{i}\left(m,n\right)$ is the complex data of the (m,n)th pixel in the SAR image of *i*th channel and *i* = 1, 2. The phase responses of a moving target in the image domain [28] of the two along-track channels can be respectively represented as

(2) $$\begin{array}{l} s_{1}=a\;\text{exp}\left(j\varphi_{0}\right)\\ s_{2}=a\;\text{exp}\left[j\left(\varphi_{0}+\frac{2\pi dv_{r}}{\lambda v_{a}}\right)\right] \end{array},$$

where the a and $\varphi_{0}$ is constant amplitude and phase, d is the channel spacing, λ is the wavelength, $v_{a}$ is the platform velocity, and $v_{r}$ is the target’s radial velocity. Then the DPCA outputs of a moving target can be expressed as

(3) $$s_{\text{DPCA}}=\left|s_{1}-s_{2}\right|=\left|a\;\text{sin}\left(\frac{\pi dv_{r}}{\lambda v_{a}}\right)\right|$$

In ideal situation, due to the existence of radial velocity, the DPCA outputs of a moving target is not zero, while that of the stationary clutter equals to approximately zero. Then the moving target can be detected by the subsequent CFAR processing after the DPCA processing. If the scene is homogeneous with Rayleigh distribution, the CA-CFAR processing can be implemented. Assume the average clutter intensity after DPCA around the moving target is σ, then the threshold T can be obtained by

(4) $$T=\sqrt{-\frac{4}{\pi }\sigma^{2}\;\text{ln}\;P_{fa}}=k\sigma ,$$

where $P_{fa}$ is the false alarm probability. If $s_{\text{DPCA}}>T=k\sigma$, the target can be detected. However, notice that the detection probability is not only related the target’s radial velocity $v_{r}$, but also proportional to the target’s original amplitude a, i.e., if the SCR

(5) $$\chi_{\text{SCR}}=\frac{a}{\sigma }>\frac{k}{\left|\text{sin}\left(\pi dv_{r}/\left(\lambda v_{a}\right)\right)\right|},$$

the target can be detected. That is, for a target with certain radial velocity, the higher SCR $\chi_{\text{SCR}}$ it has, the easier it to be detected. On the other hand, the weak target with relative original amplitude is hard to be detected by the following CFAR processing. Therefore, the river surfaces is not easy to be detected by the conventional DPCA because the $\chi_{\text{SCR}}$ is low related to the small a.

### 2.2. RR-DPCA Processing

In order to overcome the shortcoming of DPCA, a novel RR-DPCA processing is proposed in this article by jointly utilizing the DPCA outputs and the multi-look image as

(6) $$\xi =\frac{\left|x_{1}-x_{2}\right|}{\left[E\left(\left|x_{1}\right|\right)+E\left(\left|x_{2}\right|\right)\right]/2},$$

where the $\left|x_{1}-x_{2}\right|$ in the numerator is the DPCA outputs, and the $E\left(\left|x_{i}\right|\right),i=1,2$ in the denominator is the amplitude-valued multi-look image of *i*th channel and can be obtained by

(7) $$E\left(\left|x_{i}\left(m,n\right)\right|\right)=\frac{1}{L^{2}}\sum_{p=-L}^{L}\sum_{q=-L}^{L}\left|x_{i}\left(m+p,n+q\right)\right|$$

That is, the pixel (m,n) in the multi-look image is obtained by averaging the neighboring pixels intensity in a square solid-stencil window centered on $x_{i}\left(m,n\right)$, and the size of the window is L×L.

For the moving targets, if the size of window accords with the size of them in the image domain, their DPCA residue will be normalized with their average energy based on the division operator, then the targets’ RR-DPCA can be expressed as

(8) $$\xi_{T}=\left|\text{sin}\left(\frac{\pi dv_{r}}{\lambda v_{a}}\right)\right|,$$

From Equation (8), it is shown that the RR-DPCA of a moving target is mainly proportional to its radial velocity. That is, a target with larger radial velocity has larger RR-DPCA and easy to be detected, accordingly. As we know, a ground moving target has a shift in the image domain [28] because of its radial velocity, so the pixels of a target may also contain clutter. However, the rivers and oceans have large size and their velocity are relatively small, so the shift phenomenon is not obvious, and their pixels in the image domain can be regard as the components without interference of strong ground clutter. Therefore, the rivers can be detected by the RR-DPCA method as long as their radial velocity is large enough, no matter what the level of their original amplitude a is.

For the background ground clutter, it is known that the clutter with larger amplitude have smaller interferometric phase fluctuation, while the clutter with smaller amplitude have larger interferometric phase fluctuation [13]. Therefore, the ground clutter with larger amplitude is easy to be suppressed by RR-DPCA, due to their small interferometric phase as well as the cancellation operator in numerator of Equation (6). The clutter with smaller amplitude may have large residue after DPCA processing as Equation (1), while the outputs of the RR-DPCA is small due to the large denominator, denoting the average intensity of ground clutter. Therefore, the RR-DPCA processing can suppress the ground clutter well. Subsequently, the clutter statistic distribution of RR-DPCA processing will be discussed to demonstrate the clutter suppress performance.

## 3. RR-DPCA Performance Analysis

### 3.1. Clutter Statistical Distribution Model of RR-DPCA

In order to realize the CFAR processing based on RR-DPCA, its statistical distribution will be analyzed in this section. Without loss of generality, the homogeneous clutter is discussed here for simplicity. As long as the image resolution is not too high there are a large number of backscattering components in a single pixel, and the in-phase and quadrature components of the echoes are independent and identically Gaussian distributed according to the central limit theorem. Assume the variances of them are $\sigma_{i},i=1,2$, where *i* denotes the *i*th channel, it is easy to known that the DPCA outputs will obey Rayleigh distribution. The mean value of DPCA, i.e., the numerator of Equation (6) can be expressed as

(9) $$\mu_{\text{DPCA}}=\sqrt{\frac{\pi }{2}\left(\sigma_{1}^{2}+\sigma_{2}^{2}-2\rho \sigma_{1}\sigma_{2}\right)},$$

where ρ is the correlation coefficient magnitude between the two SAR images. For Equation (6), $E\left(\left|x_{i}\right|\right),i=1,2$ is the expectation of the image magnitude so the denominator of Equation (6) is a constant for the homogeneous clutter in a window, therefore, the RR-DPCA of homogenous background still obey Rayleigh distribution. As the SAR image magnitude obeys Rayleigh distribution, its expectation can be expressed as

(10) $$E\left(\left|x_{i}\right|\right)=\sqrt{\frac{\pi }{2}}\sigma_{i},\;i=1,2$$

From Equations (6), (9) and (10), the expectation of RR-DPCA can be expressed as

(11) $$\mu_{\text{RR-DPCA}}=\frac{2\sqrt{\left(\sigma_{1}^{2}+\sigma_{2}^{2}-2\rho \sigma_{1}\sigma_{2}\right)}}{\sigma_{1}+\sigma_{2}}$$

**Figure 1:**
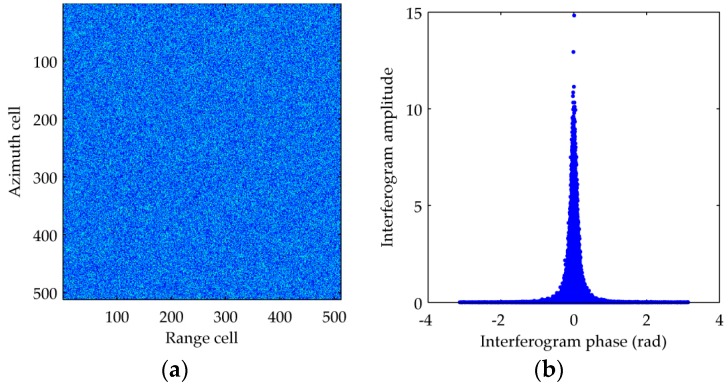

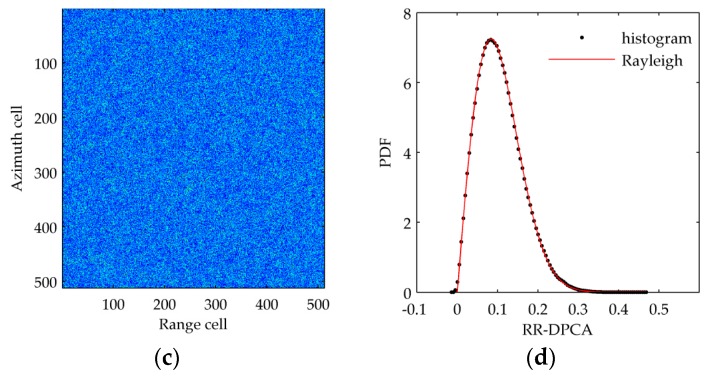
Clutter statistical distribution analysis. (**a**) 1th channel clutter image; (**b**) Interferogram amplitude and phase; (**c**) RR-DPCA image; (**d**) RR-DPCA amplitude histogram.

Next we will use simulated data to demonstrate the effectiveness of Equations (10) and (11). Firstly, the homogenous clutter of two channels are generated based on Doppler distributed clutter (DDC) model [29], and the mean value and variance of in-phase and quadrature components of clutter is zero and $\sigma_{1}=\sigma_{2}=1/\sqrt{2}$, respectively. Figure 1a shows the simulated clutter amplitude of 1th channel with the size of 512 × 512 pixels. It can be calculated that the correlation coefficient magnitude between the two channels’ clutter is 0.9945. The relationship between interferogram amplitude and phase is given in Figure 1b, which satisfies the property verified in [13]. The RR-DPCA outputs of the simulated data can be obtained as Equation (6), and the size of window to calculate the multi-look image is 9 × 9. The RR-DPCA image is shown in Figure 1c, and its histogram and the estimated Rayleigh PDF are shown in Figure 1d. It can be found that the statistics of RR-DPCA fits Rayleigh distribution very well. Furthermore, in Table 1 the theoretical and measured mean values and variances of the original clutter, DPCA outputs and RR-DPCA outputs are given, respectively. It is shown that all the errors of them are small, therefore, in the homogeneous clutter background the CA-CFAR can be implemented for the RR-DPCA based on Rayleigh distribution. It is worth mentioning that, this paper only discussed the RR-DPCA statistical distribution for homogeneous clutter, but the proposed method can also be used in heterogeneous clutter. For these cases, the CFAR processing should be changed accordingly for more complicated statistical distributions.

### 3.2. Influence of the Window Size

**Figure 2:**
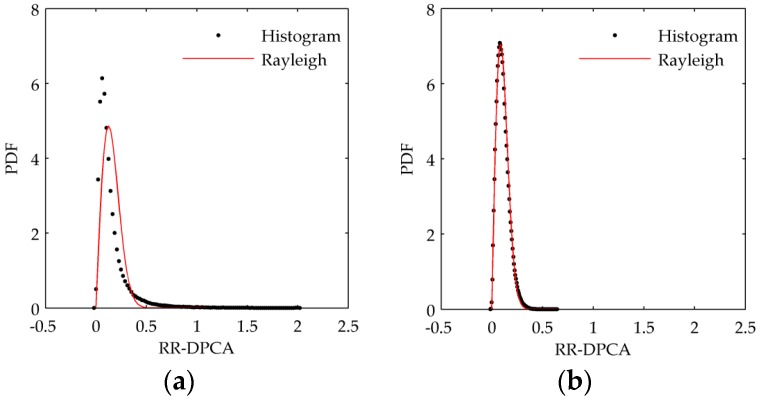

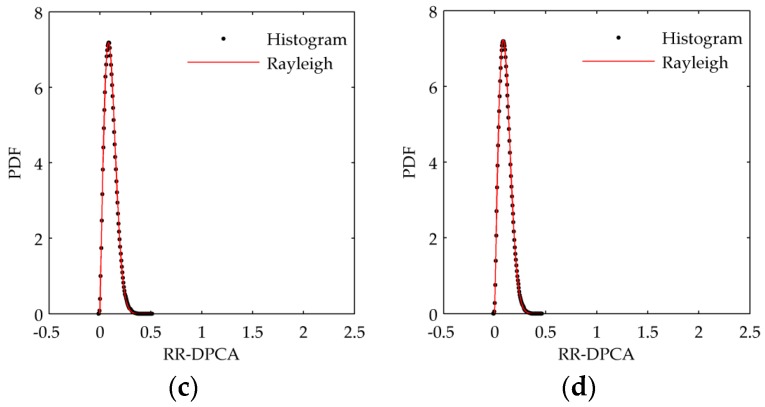
RR-DPCA histogram and its estimated PDF versus the size of window. (**a**) *L* = 1; (**b**) *L* = 3; (**c**) *L* = 5; (**d**) *L* = 7.

**Figure 3:**
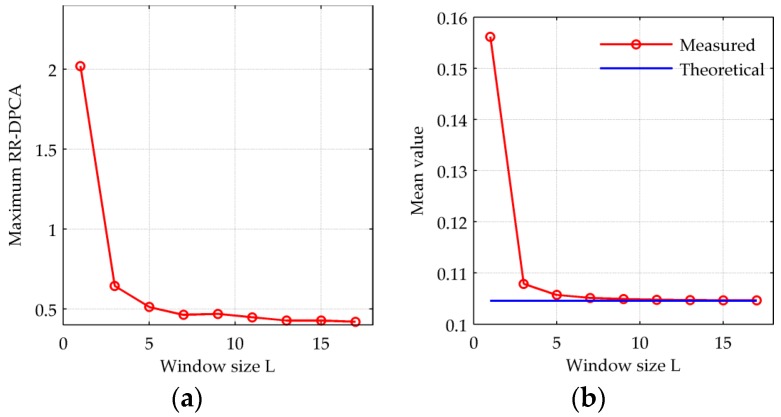
RR-DPCA versus the size of window. (**a**) Maximum RR-DPCA value versus *L*; (**b**) Maximum RR-DPCA value versus *L*.

Since the multi-look image generation is a key step for the proposed RR-DPCA method as Equation (6), it is needed to analyze the influence of window size for multi-look processing. Figure 2 shows the histogram of RR-DPCA and the estimated Rayleigh PDF versus the window size. The window size *L* in Figure 2a–d are 1, 3, 5 and 7, respectively. When *L* = 1, i.e., the denominator in Equation (6) is the sum of two origin SAR images without multi-look processing, and the statistical distribution of RR-DPCA cannot fit the Rayleigh distribution at all and has serious tailing, which will aggravate the false alarm rate. When *L* > 1, the statistical distribution of RR-DPCA can fit the Rayleigh distribution well. As the size of window increasing, the tailing is alleviating, i.e., the clutter can be suppressed well by the RR-DPCA processing with *L* > 1. Furthermore, the maximal RR-DPCA value and mean RR-DPCA value versus the size of window are given in Figure 3a,b, respectively. The maximum RR-DPCA value indicates the bigger maximum RR-DPCA value is, the more serious tailing is. When *L* > 1, the maximum RR-DPCA value decreases quickly. When *L* surpass 9, it tends to be stable. The similar change tendency can be found in Figure 3b for the mean value, when *L* > 9, the measured mean value is extremely close to the theoretical value. Therefore, in the practice, the window size *L* should be larger than 1, but don’t need to be too large, and it is nice to fit the size of detected target in image domain.

### 3.3. Flowchart of the RR-DPCA Based Method

**Figure 4:**
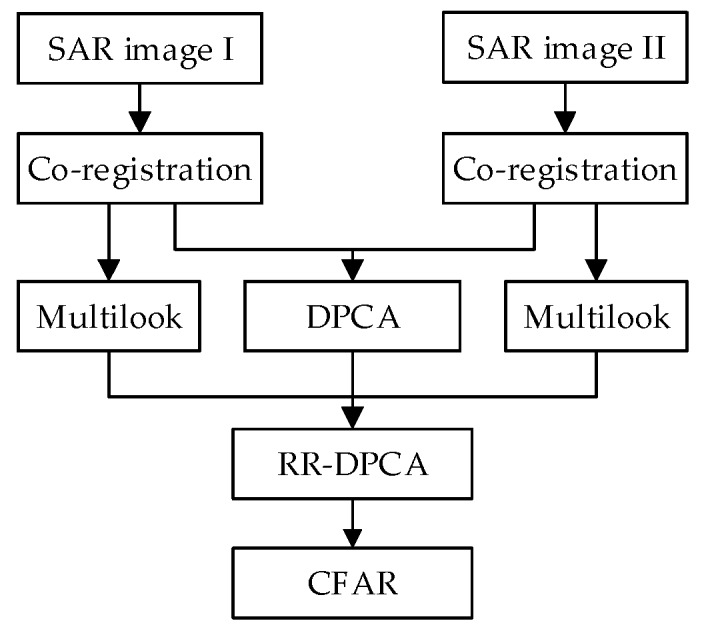
Flowchart of proposed method based on RR-DPCA.

From the analysis above, the proposed RR-DPCA method for GMTI can be summarized in Figure 4 with the following steps.

(Step 1) Focusing the two channel echoes using a SAR imaging algorithm. Then DPCA can be implemented after co-registration between the two SAR images.
(Step 2) Calculating the multi-look images of the two channel by Equation (7). The size of window should be larger than 1, but don’t need to be too large.
(Step 3) Acquiring the RR-DPCA jointly utilizing the DPCA outputs and the multi-look images by Equation (6).
(Step 4) The CFAR is applied for RR-DPCA to accomplish the target detection. Under the homogenous clutter background, the CA-CFAR can be implemented based on Rayleigh distribution. Notice that the window in CFAR is hollow-stencil, whereas that in the calculation of multi-look SAR image is solid-stencil.

## 4. Experiments Results

### 4.1. Simulated Data

#### 4.1.1. Scene Simulation

**Figure 5:**
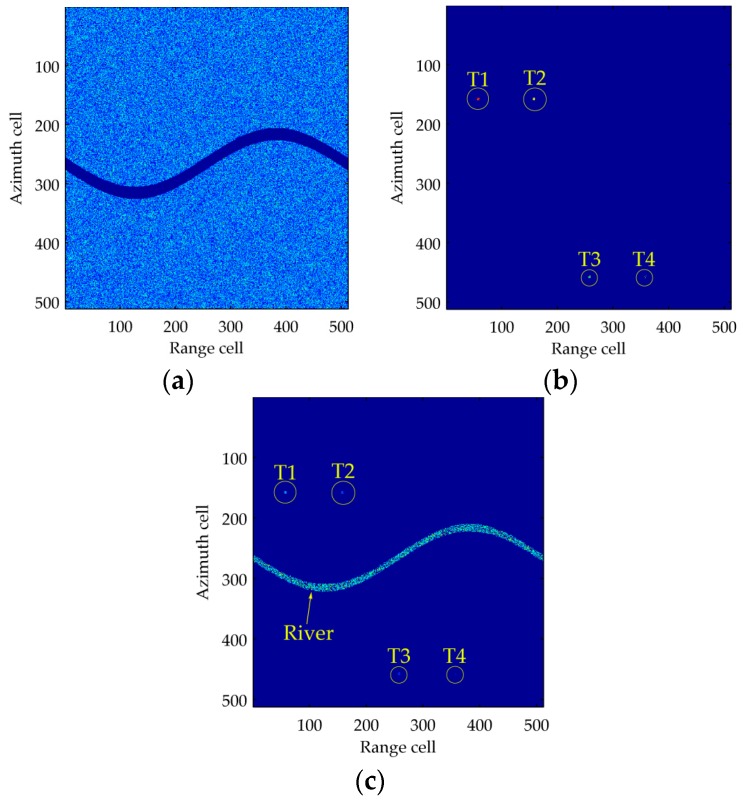
The results of the proposed RR-DPCA method based on simulated data. (**a**) 1th channel SAR image; (**b**) CFAR result of DPCA; (**c**) CFAR result of RR-DPCA.

In this section, we will demonstrate the target detection performance of the proposed method based on simulated data. The system parameters are carrier frequency 10 GHz, platform velocity 120 m/s, carrier frequency 1 GHz and channel spacing 0.5 m. The clutter of two channels are generated based on DDC model, four moving targets and a river produced by simulation are added into the two SAR images. The motion parameters are given in Table 2, the four targets have the same size 4 × 4 pixels and velocity 3 m/s, while their SCR is different. A 20 pixels wide river streams from near range to the further with a velocity 1 m/s, the SCR of it is −20 dB, i.e., its intensity is far smaller than the clutter. The clutter-to-noise ratio in the whole scene is 20 dB. The 1th channel SAR image is shown in Figure 5a, it can be found that the four targets are all submerged in the clutter and invisible, while the river pixels contain no clutter and are visible. Figure 5b is the CFAR result of the DPCA, the four targets are all detected, and however the river is missing. Figure 5c shows the CFAR result of RR-DPCA. The size of window to calculate the multi-look image is 9 × 9, and the false alarm probability is set as 10^−6^. It is shown that both the targets and the river are detected based on RR-DPCA processing.

#### 4.1.2. Performance Analysis of Point Moving Targets

**Figure 6:**
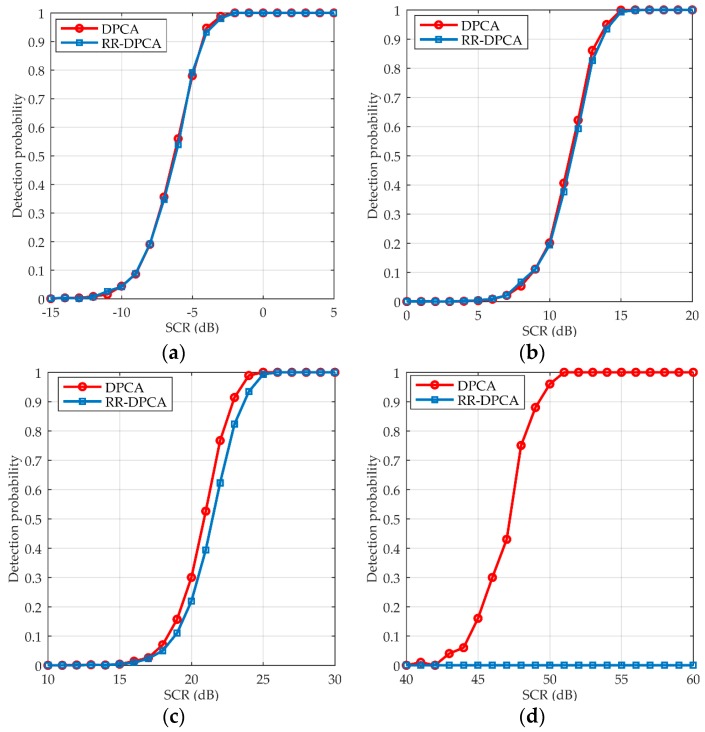
Point moving targets detection probability versus SCR with *P_fa_* = 10^−6^. (**a**) *v_r_* = 3.6 m/s; (**b**) *v_r_* = 0.3 m/s; (**c**) *v_r_* = 0.1 m/s; (**d**) *v_r_* = 0.05 m/s.

In order to analyze the detection performance for point moving targets [30], the detection probability of DPCA and RR-DPCA versus SCR is shown in Figure 6. The system and clutter parameters are the same as Section 4.1.1, it can be calculated that the blind velocity is 7.2 m/s. The target size is set as 1 × 1 pixel to simulate a point moving target, and the window size to calculate the multi-look image is 9 × 9. The target’s velocity in Figure 6a–d are 3.6 m/s, 0.3 m/s, 0.1 m/s and 0.05 m/s, respectively. When SCR increases with step 1 dB, the detection probability is counted by 1000 Monte Carlo trials with *P_fa_* = 10^−6^. It can be seen from Figure 6 that DPCA and RR-DPCA almost have the same detection performance when the velocity is large, even when $v_{r}=0.3\;m/s$. With the velocity further decreasing, the detection performance of RR-DPCA will be poorer than DPCA. When velocity is smaller than 0.05 m/s, the RR-DPCA cannot detect the target even if it has very high SCR, while DPCA still can detect it by the high SCR. Therefore, it can be concluded that DPCA and RR-DPCA almost have the same detection performance in the most range of velocity of point moving targets, and RR-DPCA cannot detect the target with very small velocity, e.g., *v_r_* = 0.05 m/s, no matter what SCR it has, while DPCA can detect it with a rather high requirement of SCR about 50 dB. However, in practice, so high SCR of a moving target is rare, and the minimum detectable velocity of RR-DPCA is small enough for most application scenarios.

### 4.2. Real Data

**Figure 7:**
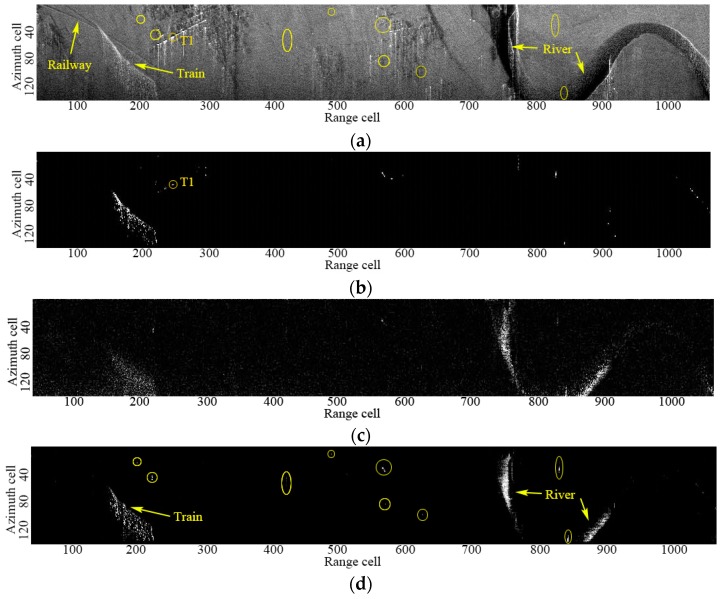
GMTI and CFAR results of different methods. (**a**) SAR image of the 1th channel; (**b**) Outputs of DPCA; (**c**) Outputs of ATI; (**d**) Outputs of RR-DPCA.

In this section, we will demonstrate the performance of the proposed RR-DPCA method by using real airborne dual-channel SAR data. The main system parameters are given as carrier frequency 9.6 GHz, bandwidth 18 MHz, sampling frequency 20 MHz, platform velocity 112 m/s, pulse repetition frequency 833 Hz and channel spacing 0.4 m. Figure 7a shows the SAR image of the 1th channel with resolution 7.5 m × 7.5 m (range × azimuth) and size of 1024 × 146 pixels. The horizontal axis and the vertical axis are the directions of range and azimuth, respectively. It can be observed that some vegetation, shrubby and strong building fields in the whole imaging scene. A railway and a defocused train can be also observed in about 200th range cell, and a river is located at the far range. The CFAR result with *P_fa_* = 10^−6^ based on DPCA is shown in Figure 7b. It is shown that the train and some other point moving targets are detected. However, the river surface is missing due to its too weak amplitude. The detection result by ATI with a threshold 1.5 is shown in Figure 7c, it can be seen that although the large-sized river can be detected, the train is not clear and many false-alarms are caused in the homogeneous clutter area. Obviously, neither ATI nor DPCA can detect the point moving targets and distributed moving targets, simultaneously.

**Figure 8:**
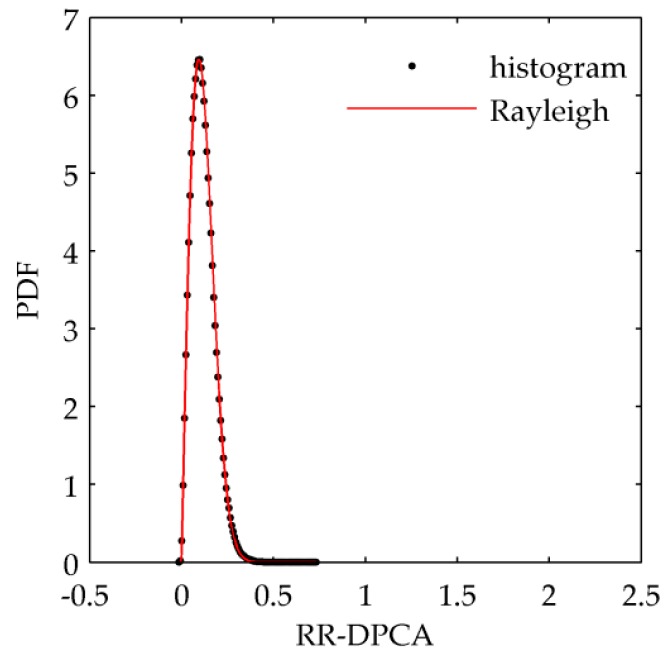
RR-DPCA histogram and estimated Rayleigh PDF of the real data.

Figure 8 shows the fitting results of Rayleigh distribution for the RR-DPCA of the homogeneous area from 350 range cell to 550 range cell. The window size to calculate the multi-look image is 11 × 11. Clearly, the Rayleigh distribution can fit the RR-DPCA well for the real data. Therefore, the CFAR threshold can be calculated by Equation (4), and the CFAR results of RR-DPCA are shown in Figure 7d. It is shown the train and the weak river surface are well detected by the proposed RR-DPCA method. Furthermore, some weak point moving targets are marked with yellow circles, and the same location are marked with yellow circles in Figure 7a, it is shown that these weak point moving targets are all invisible in the original SAR image, which demonstrate the good detection performance of the proposed RR-DPCA method for weak point moving targets. Of course, as the analysis in Section 4.1.2, a point moving target with very small velocity may be not detected via RR-DPCA, while it can be detected via DPCA if it has rather high SCR. For example, the detected target marked with “T1” in Figure 7b has a velocity 0.024 m/s and SCR 33.6 dB in the original SAR image domain, whereas it is not detected in Figure 7d. However, from the same location in Figure 7a marked with “T1”, we don’t know if T1 is a moving target, its slight interferometric phase may be caused by the channel imbalance.

## 5. Conclusions

This paper proposes a novel RR-DPCA method for ground moving target indication of dual-channel SAR, which can well detect the point moving targets and distributed moving targets like rivers simultaneously. As the rivers have low SCR in the image domain due to the specular scattering, the conventional DPCA processing is normally difficult to detect them under the strong ground clutter background. The RR-DPCA method jointly utilizes DPCA outputs and multi-look image to improve the SCR of weak river surfaces. Then the CA method can be applied for the subsequent CFAR target detection on the homogenous background. Finally, the results of both simulated and real data are provided to demonstrate the effectiveness of the proposed RR-DPCA method. 

## Figures and Tables

**Table 1:** Theoretical and measured mean value and variance.

Statistics	Value	$\left|x_{1}\right|$	$\left|x_{2}\right|$	$z_{\text{DPCA}}$	ξ
Mean value	Theoretical	0.8862	0.8862	0.0929	0.1049
	Measured	0.8854	0.8852	0.0926	0.1057
	Error	0.0008	0.0010	0.0003	0.0008
Variance	Theoretical	0.2146	0.2146	0.0024	0.0031
	Measured	0.2139	0.2139	0.0023	0.0032
	Error	0.0007	0.0007	0.0001	0.0001

**Table 2:** Targets and river parameters.

Value	Target 1	Target 2	Target 3	Target 4	River
Size (pixels)	4 × 4	4 × 4	4 × 4	4 × 4	20 width
Velocity (m/s)	3	3	3	3	1
SCR (dB)	0	−2.5	−5	−7	−20
